# Comparative Investigation of the Efficacy of Three Different Adsorbents against OTA-Induced Toxicity in Broiler Chickens

**DOI:** 10.3390/toxins7041174

**Published:** 2015-04-03

**Authors:** Jelena Nedeljković-Trailović, Saša Trailović, Radmila Resanović, Dragan Milićević, Milijan Jovanovic, Marko Vasiljevic

**Affiliations:** 1 Faculty of Veterinary Medicine, University of Belgrade, Bulevar oslobodjenja 18, 11000 Belgrade, Serbia; E-Mails: sasa@vet.bg.ac.rs (S.T.); radar@vet.bg.ac.rs (R.R.); milijan@vet.bg.ac.rs (M.J.); 2 Institute of Meat Hygiene and Technology, Kacanskog 13, 11040 Belgrade, Serbia; E-Mail: dragan@inmesbgd.com; 3 Patent Co. d.o.o, 24211 Mišićevo, Serbia; E-Mail: marko.vasiljevic@patent-co.com

**Keywords:** ochratoxin A, adsorbents, performance, pathohistological examination

## Abstract

The aim of our study was to determine the efficacy of three different adsorbents, inorganic (modified zeolite), organic (esterified glucomannans) and mixed (inorganic and organic components, with the addition of enzymes), in protecting broilers from the toxic effects of ochratoxin A in feed. Broilers were fed diets containing 2 mg/kg of ochratoxin A (OTA) and supplemented with adsorbents at the recommended concentration of 2 g/kg for 21 days. The presence of OTA led to a notable reduction in body weight, lower weight gain, increased feed conversion and induced histopathological changes in the liver and kidneys. The presence of inorganic, organic and mixed adsorbents in contaminated feed only partially reduced the negative effects of OTA on the broiler performances. Broilers that were fed with adsorbent-supplemented feed reached higher body weight (17.96%, 19.09% and 13.59%), compared to the group that received only OTA. The presence of adsorbents partially alleviated the reduction in feed consumption (22.68%, 12.91% and 10.59%), and a similar effect was observed with feed conversion. The applied adsorbents have also reduced the intensity of histopathological changes caused by OTA; however, they were not able to prevent their onset. After the withdrawal of the toxin and adsorbents from the feed (21–42 days), all previously observed disturbances in broilers were reduced, but more remarkably in broilers fed with adsorbents.

## 1. Introduction

Mycotoxins are secondary toxic metabolites produced by different species of molds [1,2]. They usually enter the bodies of humans and animals through contaminated food/feed infested with spores, conidia or mycelial fragments. Therefore, in most cases, intoxications or mycotoxicoses occurs through oral intake. Their etiological relation to food also implies possible large-scale proportions of intoxications. Accordingly, contamination of animal feed with mycotoxins represents a worldwide problem in veterinary and human medicine [3]. Mycotoxins that originate from the *Aspergillus* spp*.* are a very significant group of biological toxins considering the effects that they induce. Considering the prevailing climate in southeastern Europe, the presence of the *Aspergillus* and *Penicillium* molds and the toxin content in feeds and food, ochratoxin A (OTA), is highly significant. Lesions of the acute ochratoxicosis in poultry are primarily found in the kidneys and in the liver [4,5,6]. Nephropathies caused by mycotoxins are followed by degeneration of the tubules, joined with interstitial fibrosis, as well as predominant changes and damage of tubular activity [7]. Many authors reported that OTA could provoke different pathohistological changes in kidney and liver [8,9,10]. During a histopathological examination of the broilers’ liver, vascular degeneration, hyperemia and precapillary edema of the Kupffer cells with a perivascular infiltration were determined. Furthermore, degenerative changes in the proximal renal tubular cells were noticed in the kidneys of treated broilers. The cells were blurred and swollen with karyopyknosis and karyolysis. Finally, a mononuclear cell infiltration was detected in the interstitium of the kidney [11].

The most common method for the reduction or elimination of the harmful effects of mycotoxins in clinical practice is the use of adsorbents [12,13]. Adsorbents are substances that are not resorbed by the intestines, but have the capability for specific binding of natural chemical substances, which prevent their absorption. In clinical practice, the most frequently used adsorbents are inorganic products (activated carbon, sodium-calcium hydrated aluminosilicate, bentonite, various clays and aluminosilicates-zeolites) [14]. However, in recent years, the efficiency of organic adsorbents, especially modified mannan oligosaccharides, isolated from the inner layer of the cell wall of yeasts, has been explored. They have a distinct ability to adsorb mycotoxins, which is of special clinical significance [15,16]. On the other hand, biological detoxification of mycotoxins can be defined as biotransformation, *i.e.*, enzyme-facilitated degradation, accomplished through the activity of either the whole cell of the microorganism or the enzyme system of the individual cell. This relatively new approach of mycotoxin detoxification is more often applied to non-polar toxins, while a number of studies are focused on the application on polar mycotoxins, as well.

Considering that, to the best of our knowledge, there are no comparative studies of the effectiveness of two or more different types of adsorbents added to the feed in relation to ochratoxin A toxicosis, we have found that it is important to investigate this on broiler chickens in controlled conditions.

## 2. Results

### 2.1. Broiler Performance

Changes in body weight of broilers at various stages of the experiment are shown in Table 1, while the average daily gain, the average daily feed intake and feed conversion during the investigation are shown in Table 2 and Table 3.

Results from Table 1 show that the highest body weight was achieved in the broilers from the control group (C), followed by broilers from groups that were given feed supplemented with three different adsorbents Minazel, Mycosorb and Mycofix (Mz, Ms and Mf), without the addition of the toxin. Differences in body weights between the control (C) and the experimental group, which was given 2 mg/kg of OTA supplemented with 0.2% of inorganic adsorbent (Experimental Group II (E-II)), were significant (*p <* 0.01) and highly significant (*p <* 0.001) on the 21st and 42nd day of the study. There were no significant differences in the production results (*p >* 0.05) between the group to which only OTA was administered and the group that was given OTA with 0.2% of Mycofix (E-IV). During the entire trial, no statistically-significant differences were observed between the body weight of broilers from the control group (C) and the groups that received three different adsorbents in the feed.

During the observation period, after withdrawal of OTA and the adsorbents from the feed (21st to 42nd day), the average values of the body weight of broilers from the experimental groups were almost equal. At the end of the observation, differences in weight between E-I, E-II, E-III and E-IV did not exceed 100 g. However, these values were significantly lower (*p <* 0.001) than the average body weight of broilers from the control group (C) and also the groups previously treated only with adsorbents (Mz, Ms, Mf). Broilers from the control group (C), as well as the groups that were fed mixtures supplemented with the adsorbents, for 21 days, reached a body weight within the limits of the technological standards on Day 42.

During the first three weeks of the trial, slightly higher daily weight gain was observed in broilers that received feed supplemented with adsorbents (Mz, Ms, Mf), compared to the control group (C). Higher weight gain was recorded from Day 14 to Day 21 in broilers that received the inorganic adsorbent only (*p <* 0.001). However, by the end of the trial, this difference disappeared, resulting in the highest weight gain of the control broilers (Table 2).

Daily feed intake during the trial is shown in Table 3. Broilers from the control groups (C, Mz, Ms, Mf) consumed the usual amounts of feed. The groups that received feed with 0.2% of the adsorbent consumed somewhat larger amounts of feed in comparison to the control group. The experimental groups consumed lower amounts of feed (almost 50% less) compared to the control group and the other experimental groups that were given 0.2% of various adsorbents in the feed. The feed conversion ratio was lower in broilers from the control group (2.12 kg) compared to the experimental groups. The highest conversion was achieved in the group that received only 2 mg/kg of OTA in feed (2.37 kg), while the broilers from other experimental groups, which were given feed supplemented with OTA and 0.2% of the adsorbent, achieved somewhat lower conversion compared to the E-I group. Broilers from the control group achieved a slightly higher conversion compared to the groups that received 0.2% of different adsorbents in feed (Table 4).

**Table 1:** Average body weight of broilers during the study (g), (mean ± SE). E-I, ochratoxin A (OTA) 2 mg/kg feed; E-II, OTA 2 mg/kg feed + 0.2% Mz; E-III, OTA 2 mg/kg feed + 0.2% Ms; E-IV, OTA 2 mg/kg feed + 0.2% Mf; C, control, without toxin and adsorbents in feed; Mz, Ms, Mf, 0.2% of adsorbents in feed. The period from 1 to 21 days involves feeding with the addition of toxin and adsorbents, while the period from 21 to 42 days involves feeding with the commercial feed only, without toxin and adsorbents.

Days of the Study						
Group	(*n*)	1	7	14	21	42
**E-I**	20	48.26 ± 0.63	113.2 ± 2.82	184.8 ± 3.00 ^aaa^	293.8 ± 9.83 ^aaa^	1,080 ± 71.5 ^aaa^
**E-II**	20	48.30 ± 0.85	118.5 ± 3.17	219.6 ± 3.83 ^a,b^	358.1 ± 11.71 ^aa,b^	1,174 ± 31.09 ^aaa^
**E-III**	20	48.31 ± 0.82	127.3 ± 2.86 ^b^	220.4 ± 10.01 ^a,b^	362.8 ± 13.81 ^aa,b^	1,158 ± 62.89 ^aaa^
**E-IV**	20	48.27 ± 0.66	120.7 ± 3.60	206.8 ± 10.17 ^a^	340.0 ± 18.25 ^aaa^	1,102 ± 56.97 ^aaa^
**C**	20	48.24 ± 1.24	123.5 ± 4.87	262.8 ± 8.88	458.3 ± 18.52	1,796 ± 39.88
**Mz**	20	48.30 ± 0.81	142.8 ± 3.68 ^bbb,ccc,ee^	296.0 ± 9.98 ^bbb,ccc,ddd,eee^	533.3 ± 12.42 ^bbb,ccc,dd,eee^	1842 ± 42.99 ^bbb,ccc,ddd,eee^
**Ms**	20	48.29 ± 0.68	136.9 ± 3.69 ^bbb,c^	271.3 ± 11.47 ^bbb,cc,dd,eee^	429.4 ± 17.19 ^bbb,ee,ff^	1,736 ± 30.80 ^bbb,ccc,ddd,eee^
**Mf**	20	48.32 ± 1.01	133.5 ± 4.74 ^bb^	283.0 ± 10.83 ^bbb,ccc,ddd,eee^	458.3 ± 15.14 ^bbb,cc,dd,eee^	1,761 ± 35.02 ^bbb,ccc,ddd,eee^

^a^ Statistically-significant difference compared to the control (a = *p* < 0.05; aa = *p* < 0.01; aaa = *p* < 0.001). ^b^ Statistically-significant difference compared to the E-I (b = *p* < 0.05; bb = *p* < 0.01; bbb = *p* < 0.001). ^c^ Statistically-significant difference compared to the E-II (c = *p* < 0.05; cc = *p* < 0.01; ccc = *p* < 0.001). ^d^ Statistically-significant difference compared to the E-III (dd = *p* < 0.01; ddd = *p* < 0.001). ^e^ Statistically-significant difference compared to the E-IV (ee = *p* < 0.01; eee = *p* < 0.001). ^f^ Statistically-significant difference compared to the Mz (ff = *p* < 0.01).

**Table 2:** Average daily gain of broilers during the study (g), (mean ± SE). E-I OTA, 2 mg/kg feed; E-II, OTA 2 mg/kg feed + 0.2% Mz; E-III, OTA 2 mg/kg feed + 0.2% Ms; E-IV, OTA 2 mg/kg feed + 0.2% Mf; C, control, without toxin and adsorbents in feed; Mz, Ms, Mf, 0.2% of adsorbents in feed. The period from 1 to 21 days involves feeding with the addition of toxin and adsorbents, while the period from 21 to 42 days involves feeding with the commercial feed only, without toxin and adsorbents.

Days of the study						
Group	(*n*)	1–7	7–14	14–21	21–42	1–42
**E-I**	20	9.28 ± 0.32	10.67 ± 0.13 ^aaa^	15.43 ± 0.98 ^aaa^	37.17 ± 3.57 ^aaa^	24.54 ± 1.69 ^aaa^
**E-II**	20	10.0 ± 0.34	14.59 ± 0.23 ^aa^	20.16 ± 1.29 ^aa^	39.78 ± 1.25 ^aaa^	26.89 ± 0.73 ^aaa^
**E-III**	20	11.28 ± 0.29 ^b^	13.66 ± 0.94 ^aaa^	20.93 ± 0.69 ^a,b^	40.56 ± 2.52 ^aaa^	26.41 ± 1.47 ^aaa^
**E-IV**	20	10.35 ± 0.42	11.25 ± 0.89 ^aaa,cc^	19.54 ± 1.26 ^aaa^	41.57 ± 1.87 ^aaa^	25.06 ± 1.35 ^aaa^
**C**	20	10.74 ± 0.54	19.90 ± 0.70	27.93 ± 1.49	64.55 ± 1.89	42.12 ± 0.88
**Mz**	20	13.49 ± 0.41 ^aa,bbb,ccc,d,eee^	21.89 ± 1.58 ^bbb,ccc,ddd,eee^	34.03 ± 2.47 ^bbb,ccc,ddd,eee^	61.54 ± 1.96 ^bbb,ccc,ddd,eee^	42.88 ± 1.03 ^bbb,ccc,ddd,eee^
**Ms**	20	12.65 ± 0.44 ^bbb,cc,ee^	19.31 ± 1.24 ^bbb,cc,ddd,eee^	22.58 ± 0.96 ^aa,gg^	62.64 ± 1.43 ^bb,ff^	40.11 ± 0.76 ^bbb,ccc,ddd,eee^
**Mf**	20	12.17 ± 0.54 ^bbb,ccc,ddd,eee^	21.35 ± 0.97 ^bbb,ccc,ddd,eee^	27.77 ± 0.99	62.03 ± 1.83 ^bb,ff^	40.51 ± 0.84 ^bbb,ccc,ddd,eee^

^a^ Statistically-significant difference compared to the control (a = *p* < 0.05 aa = *p* < 0.01 aaa = *p* < 0.001). ^b^ Statistically-significant difference compared to the E-I (b = *p* < 0.05; bb = *p* < 0.01; bbb = *p* < 0.001). ^c^ Statistically-significant difference compared to the E-II (cc = *p* < 0.01; ccc = *p* < 0.001). ^d^ Statistically-significant difference compared to the E-III (d = *p* < 0.05; ddd = *p* < 0.001). ^e^ Statistically-significant difference compared to the E-IV (ee = *p* < 0.01; eee = *p* < 0.001). ^f^ Statistically-significant difference compared to the Mz (ff = *p* < 0.01). ^g^ Statistically-significant difference compared to the Ms (gg = *p* < 0.01).

**Table 3:** Average daily feed intake (g). E-I, OTA 2 mg/kg feed; E-II, OTA 2 mg/kg feed + 0.2% Mz; E-III, OTA 2 mg/kg feed + 0.2% Ms; E-IV, OTA 2 mg/kg feed + 0.2% Mf; C, control, without toxin and adsorbents in feed; Mz, Ms, Mf, 0.2% of adsorbents in feed. The period from 1 to 2 days involves feeding with the addition of toxin and adsorbents, while the period from 21 to 42 days involves feeding only with the commercial feed, without toxin and adsorbents.

Days of the Study				
Group	1–21	21–35	35–42	1–42
**E-I**	24.32	71.93	123.20	56.67
**E-II**	32.15	89.68	144.30	70.04
**E-III**	28.43	84.95	116.89	62.01
**E-IV**	28.79	77.47	130.23	61.92
**C**	34.39	130.00	188.94	92.02
**Mz**	35.91	130.85	202.00	95.24
**Ms**	36.02	128.53	179.46	93.77
**Mf**	35.93	127.95	203.59	94.54

**Table 4:** Feed conversion ratio (FCR). E-I, OTA 2 mg/kg feed; E-II, OTA 2 mg/kg feed + 0.2% Mz; E-III, OTA 2 mg/kg feed + 0.2% Ms; E-IV, OTA 2 mg/kg feed + 0.2% Mf; C, control, without toxin and adsorbents in feed; Mz, Ms, Mf, 0.2% of adsorbents in feed. The period from 1 to 2 days involves feeding with the addition of toxin and adsorbents, while the period from 21 to 42 days involves feeding only with the commercial feed, without toxin and adsorbents.

Days of the Study				
Group	1–21	21–35	35–42	1–42
**E-I**	2.03	2.67	2.79	2.37
**E-II**	2.02	2.24	2.80	2.24
**E-III**	1.99	2.50	2.61	2.26
**E-IV**	2.02	2.22	2.75	2.23
**C**	1.87	2.24	2.66	2.12
**Mz**	1.81	2.16	2.54	2.05
**Ms**	1.80	2.12	2.10	2.03
**Mf**	1.81	2.17	2.56	2.05

### 2.2. Pathomorphological Examination

Pathomorphological examination of sacrificed broilers from Group E-I, which received feed contaminated with 2 mg/kg of OTA, revealed pale and enlarged kidneys and macroscopically visible hemorrhages in five out of 10 animals. Pale and enlarged liver of a crumbly consistency was observed in three out of 10 animals, while in one animal, a subcapsular hematoma was noted. The obtained results are in accordance with our previous results [4], as well as with those published by Kumar *et al.* [17].

Pale and enlarged kidneys without macroscopically-visible hemorrhages were observed in three out of 10 broilers from Group E-II, which received OTA supplemented with 0.2% of inorganic adsorbent (Mz). Furthermore, enlarged liver of a crumbly consistency was noted in two birds. A similar finding was seen in the other two experimental groups that received OTA with 0.2% of organic (Ms) (E-III) and mixed adsorbent (Mf) (E-IV). Macroscopic changes in the form of enlarged pale kidneys without hemorrhage were recorded in two out of 10 animals of both experimental groups. Pale and enlarged liver was observed in two out of 10, *i.e.*, one out of 10 sacrificed animals from the E-III and E-IV experimental groups. No pathoanatomical changes were observed in broilers that were fed with different adsorbents (Mz, Ms and Mf) only.

### 2.3. Pathohistological Examination

**Figure 1:**
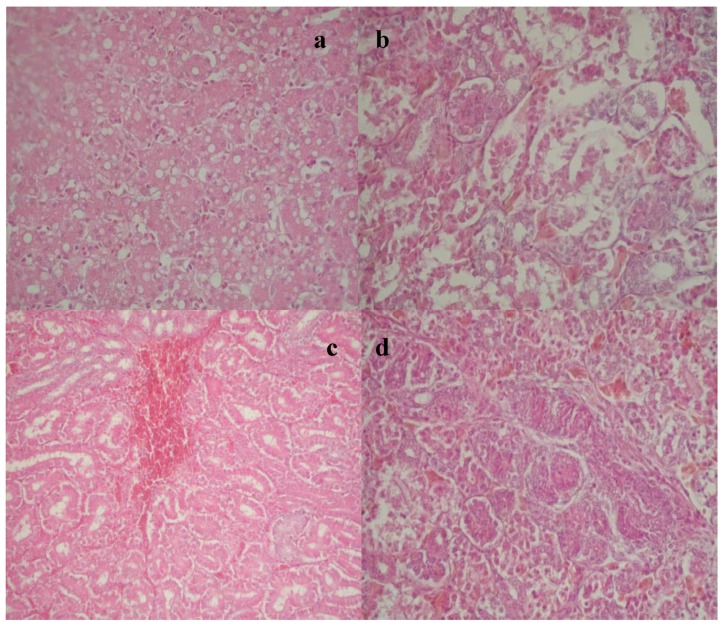
(**a**) Hepatocyte vacuolation due to the accumulation of lipid droplets, Experimental Group I (E-I); (**b**) Necrotic foci localized in tubulocytes, E-I group; (**c**) Hemorrhagic areas with massive effusion of red blood cells, E-I group; (**d**) Renal tubular cell proliferation and formation of adenoma-like structures, E-I group.

No deviation from the normal histological structure of the liver and kidneys was observed in broilers from the control group (C) and the groups that received feed supplemented with different adsorbents (Mz, Ms and Mf). Hepatocyte vacuolation was observed in broilers from the E-I group, which received feed supplemented with 2 mg/kg of OTA during three weeks, due to diffuse accumulation of lipid droplets (Figure 1a). Pathohistological examination of the kidneys revealed that renal proximal tubules were predominantly affected. Epithelial cells of renal proximal tubules were enlarged with opaque cytoplasm. A small number of necrotic foci localized in tubulocytes were also observed (Figure1b). In some birds from the E-I group, hemorrhagic areas with massive effusion of red blood cells were noted (Figure1c). Renal tubular cell proliferation and the formation of adenoma-like structures were observed in two animals from this group (Figure 1d).

**Figure 2:**
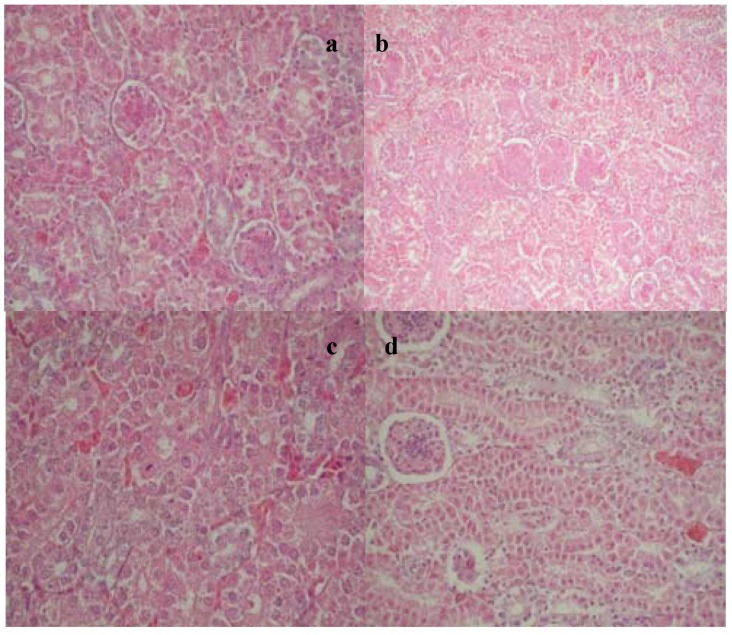
(**a**) Edema of the renal proximal tubule cells with partial tubule lumen stenosis, dystrophic changes with the appearance of apoptotic bodies, E-II group; (**b**) Proliferation of mesangial cells and capillary endothelial cells in the glomeruli, E-II group; (**c**) Regenerative changes in the tubulocytes, E-I group after the withdrawal period; (**d**) Regenerative changes in the tubulocytes and sclerotic changes in the glomeruli, E-I group after the withdrawal period.

Pathohistological examination of broilers from Group E-II, which were fed a mixture contaminated with 2 mg/kg of OTA and 0.2% of inorganic adsorbent, revealed that changes were of a lower intensity compared to the group that received only OTA. Changes were manifested in the form of focal fat accumulation within hepatocytes. In the kidneys of broilers from Group E-II, changes in the form of edema of the renal proximal tubule cells were noted, as well as vacuolation with partial tubule lumen stenosis, but without any signs of necrosis (Figure 2a). Proliferation of mesangial cells and capillary endothelial cell edema was noted in some of the birds from this experimental group, as well as hyperemia, which consequently led to an increase in glomerular volume (Figure 2b).

Fatty changes of lower intensity in the form of focal fat accumulation were recorded in the liver of broilers from the Experimental Group E-III that was fed a mixture supplemented with 2 mg/kg of OTA and organic adsorbent. In the kidneys from this experimental group, edema of the renal proximal tubule cells, as well as vacuolation, with partial stenosis of tubular lumen, were observed. Dystrophic changes with the appearance of apoptotic bodies were also noted in these cells. In some animals, the multiplication of cells in vascular loops, as well as hemorrhage in the capsule and the renal cortex was observed, as well. In broilers from Group E-IV, which were fed a mixture supplemented with 2 mg/kg of OTA and mixed adsorbent during three weeks, a discrete hemorrhage was noted in the liver. Weak hemorrhages were observed in kidneys, as well, at the border between cortex and medulla. Vacuolization and cell dystrophy of renal proximal tubules without changes in the nuclei were also found. Dystrophic changes in the form of cytoplasmic vacuolation and nucleus pyknosis were noted in the renal medullary cells.

The group that received 2 mg/kg of OTA for three weeks and that, to the end of the experiment, was fed without toxin showed discrete hemorrhage in the hepatocytes and renal medulla. Regenerative changes with mitosis in cells were observed in tubulocytes. Furthermore, glomeruli sclerotic changes were noted in the form of capsule and vascular loop thickening (Figure 2c,d). In all three experimental groups, which took feed containing 2 mg/kg of OTA with various adsorbents for three weeks, followed by mixtures without toxins and adsorbents for another three weeks, no pathohistological changes were observed in the glomeruli. Moreover, evident signs of regeneration were observed in the tubules. On the other side, pathohistological changes could not be observed in the hepatocytes of the birds from all experimental groups, after three weeks of the resting period, indicating intensive processes of cellular restitution.

## 3. Discussion

### 3.1. Broiler Performance

Production results achieved during the experiment were different in the control and experimental groups, depending on the impact of OTA and the presence or absence of various adsorbents in feed. Comparing the experimental groups with the control one, it can be seen that OTA is responsible for a negative effect on the body weight of broilers. On the 21st day of the trial, the body weight of broilers from the experimental groups (E-I, E-II, E-III and E-IV) was 35.90, 21.87, 20.84 and 25.71% lower than in the control group. At the end of the experiment, the body weight of broilers from the experimental groups was 39.87, 34.64, 35.56 and 38.65% lower than in broilers from the control group. It was also observed that the animals that were given feed supplemented with adsorbents (E-II 17.96%, E-III 19.09% and E-IV 13.59%) reached higher body weight on Day 21 compared to the group that received only OTA (E-I) (Table 1). Taking into consideration these results, it can be concluded that the adsorbents showed only partial protection against the adverse effects of OTA with respect to body weight.

Although body weight is a good indicator for the health status of the birds, average daily gain is considered to reflect the quality of feed and, especially, its safety parameters more reliably. Comparing the experimental groups with the control one in both experiments, it was noted that OTA has a negative effect on daily weight gain. Average daily gain of broilers from Day 1 to Day 21 of the experiment (E-I, E-II, E-III and E-IV) was lower compared to the control group (44.76%, 27.28%, 25.27% and 30.04%). Furthermore, in animals that were fed contaminated feed supplemented with adsorbents, higher daily gain was noted in the period from Day 1 to Day 21, compared to the group that was given only OTA (E-II 10.48%, E-III 19.49% and E-IV 14.72%). A declining trend in weight gain remained in the period after withdrawal of toxins and adsorbents.

The control group consumed the amount of feed usual for the species and age during the trial, while broilers that were given feed with 0.2% of adsorbents consumed slightly larger amounts. On the other hand, the experimental groups fed with OTA consumed lower amounts of feed compared to the control group and the experimental groups that were fed mixtures with 0.2% of various adsorbents. The presence of adsorbents in mixtures, in the period from Day 1 to Day 21, only partially alleviated the reduction in feed consumption in experimental groups (E-II 22.68%, E-III 12.91% and E-IV 10.59%) compared to the group that was fed mixtures supplemented with 2 mg/kg of OTA only.

According to our results, feed conversion, which is the measure of the interaction of growth and consumption, was different between the control and experimental groups. From Day 1 to Day 21 of the trial, in broilers from the experimental groups (E-I, E-II, E-III and E-IV), conversion was 8.55, 8.02, 6.42 and 8.02% lower compared to broilers from the control group. During the entire experiment, from Day 1 to Day 42, conversion in the experimental groups was 11.79, 5.66, 6.60 and 5.18% lower compared to the broiler chickens from the control group. These findings are consistent with the data of Chang *et al.* [18], who reported significantly worse results in the feed conversion by using only OTA in doses of 4 and 8 mg/kg for three weeks, and partially in agreement with the findings of Nedeljkovic-Trailovic *et al.* [19], due to the different timing of the administration of OTA.

Our results are in agreement with the findings of Santin *et al.* (2003). They have demonstrated that the addition of the cell walls of *Saccharomyces cerevisiae* (CWSC) to the broilers’ diet containing the OTA (0.5 mg/kg) improves the feed conversion of birds exposed to ochratoxin for 42 days. However, CWSC did not ameliorate feed intake and weight gain [20]. Santin *et al.* (2002) have previously published another research work, this time using hydrated sodium calcium aluminosilicate (HSCAS) as the adsorbent and 2 mg/kg of OTA in the broilers’ feed. The authors state that ochratoxin in the diet impaired the productivity indexes, and that HSCAS did not improve these parameters [14].

### 3.2. Pathoanatomical Examination

The autopsy of sacrificed broilers from the experimental group (E-I) that was given feed supplemented only with OTA showed clear macroscopic changes in liver and kidneys, observed in 50 and 30% of the birds. The occurrence of similar changes in kidneys and liver of broilers from the experimental group that was fed mixtures supplemented with OTA and 0.2% of the inorganic adsorbent (E-II) was less common, but with almost the same characteristics. A similar finding was noted in the other two experimental groups, which received OTA with 0.2% of organic (E-III) and mixed adsorbent (E-IV). Macroscopic changes in the form of enlarged pale kidneys without hemorrhage were recorded in 20% of animals of both experimental groups. Pale and enlarged liver was observed in 20 and 10% of sacrificed animals. Our findings are in accordance with the results of Santin *et al.* [14], who fed broilers mixtures supplemented with 2 mg/kg of OTA and 0.25% of aluminosilicate.

### 3.3. Pathohistological Examination

In broilers that were given mixtures with 2 mg/kg of OTA for three weeks (E-I), vacuolization due to diffuse accumulation of lipid droplets was observed in blurred and swollen hepatocytes. These results are in accordance with the findings of Koynarski *et al.* [11], as well as Kubena *et al.* [21], who noted hepatic lipidosis and biliary hyperplasia in the liver of the tested animals that were given feed with 1 and 2 mg/kg of OTA [22]. However, these results contradict the reports of Nedeljkovic-Trailovic *et al.* [4], who fed broilers mixtures contaminated with 1.5 mg/kg of OTA for seven days and found no pathohistological changes in the liver. This was probably due to a somewhat lower concentration of OTA than the one used in our experiment, as well as shorter exposure in comparison to our trial. This confirms the findings of most authors, who report that the intensity of pathohistological changes in the liver depends on the concentration of OTA and the duration of exposure [23,24].

Histopathological findings in which focal accumulation of lipid droplets in hepatocytes was dominant were identified in broilers from the other three experimental groups that received OTA plus 0.2% of different adsorbents (E-II, E-III and E-IV). Our results are consistent with the findings of Santin *et al.* [14], who fed broilers mixtures contaminated with 2 mg/kg of OTA and supplemented with 0.25% of inorganic adsorbent. Pathohistological examination of the hepatocytes revealed vacuolization and megalocytosis, as well as bile ductular cell hyperplasia. Three weeks after withdrawal of OTA and the adsorbents, complete recovery of hepatocytes was observed in experimental animals, and no pathohistological changes were detected. These findings are in accordance with the report of Garcia *et al.* [12]. However, to the best of our knowledge, there are no data in the available literature on comparative examination of the three adsorbents and the manifestation of their effects on alleviation of the intensity of pathohistological changes in the liver and kidneys of broilers treated with OTA.

According to Roth *et al.* [25], as well as Fuchs *et al.* [26], hepatotoxicity induced by OTA represents a consequence of its metabolism. These authors have observed in their experiments a secondary maximal concentration of OTA in the intestines and the serum of rodents, which were explained as a consequence of enterohepatic recirculation. During the transport through the liver, OTA is exposed to conjugation and excreted via bile into the intestine in the form of glucuronide and sulfate. These conjugates are subjected to hydrolysis in the lumen of the intestine by intestinal microflora and transform again into OTA and OTAα, which are reabsorbed. This specific metabolism increases systemic redistribution of OTA in different tissues and potentiates its toxicity.

Similar pathohistological changes of different intensities were observed in the kidneys of broilers from all experimental groups. All changes in the kidneys caused by ochratoxin A were primarily localized in the renal proximal tubules, this being related to the toxin metabolism. Pathohistological examination of the kidneys revealed that the changes mainly occurred in the renal proximal tubules in broilers that received feed with 2 mg/kg of OTA (E-I) during three weeks. Epithelial cells of the renal proximal tubules were blurry and swollen. Necrotic foci localized in tubulocytes were detected in a smaller number of animals. Hemorrhagic areas with a large effusion of erythrocytes were recorded in some animals of this experimental group, as well. In accordance with our results, Kumar *et al.* [17] describe the changes in renal tubule cells of broilers fed with 2 mg/kg of OTA over the period of three weeks. The changes that occurred in the kidneys were primarily localized in the renal proximal tubules, which were described as swollen. Focal interstitial nephritis with renal tubular cell degeneration was also observed. Similar pathohistological changes in the kidneys of broilers were observed by Nedeljkovic-Trailovic *et al.* [4]. Pathohistological examination of the kidneys revealed that renal proximal tubules were mainly affected in animals that were given feed contaminated with 1.5 mg of OTA/kg for seven days.

The proliferation of renal tubule cells and the formation of adenomatous structures were observed in two animals from the experimental group of our trial that was fed mixtures supplemented only with the toxin (E-I). Furthermore, collecting duct cells’ pyknotic nuclei were noted in the kidney, as well as apoptotic bodies and medullary hyperemia. These results are in accordance with the reports of Biro *et al.* [27], who observed proliferated tubulocyte cells (adenoma-like changes) in the renal parenchyma in several animals. Son *et al.* [28] reported the occurrence of renal adenomas in female Lewis and Dark Agouti DA rats, treated with 0.4 mg/kg/BW of OTA for three weeks.

Histopathological examination revealed similar changes in individual animals from all three experimental groups (E-II, E-III and E-IV). A significant difference between broilers from the group that received only OTA and the groups that received contaminated feed supplemented with 0.2% of different adsorbents confirms the findings of the necrotic changes in the tubules that were found in broilers that were given only the toxin. Comparative changes were not observed in groups that received the toxin with different adsorbents. Multiplication of the proximal tubule cells and the formation of adenoid structures were also observed in broilers that received only the toxin. However, this was not observed in other experimental groups that received feed supplemented with the toxin and the adsorbents.

Data on the efficacy of adsorbents in alleviating the histopathological changes in the kidneys that occur under the influence of OTA are scarce. Our results are consistent with the reports of Santin *et al.* [14], who examined the efficacy of aluminosilicate in alleviating the adverse effects of OTA in kidneys. These authors found that in the group of broilers that, in addition to OTA, received 0.25% of aluminosilicate, the adsorbent only partially alleviated the intensity of changes, showing no significant protection capabilities. Our results are partially consistent with the findings of Garcia *et al.* [12], who report that Zeotek added to the feed in the amount of 0.15% significantly reduced the percentage of necrotic cells in tubulocytes. Garcia *et al.* [12] report that 0.25% of Mycofix in the presence of 0.567 mg/kg of OTA led to a significant decrease in the number of necrotic cells in tubulocytes.

Histopathological examination of the kidneys revealed medullar hemorrhage in the group that received 2 mg/kg of OTA and then was fed mixtures without the toxin for three weeks. Regenerative changes with the occurrence of mitosis in the cells were noted in tubulocytes. Glomerular sclerotic changes were detected in the form of thickening of the capsule and the vascular loop. These results are consistent with the findings of Nedeljkovic-Trailovic *et al.* [4], who report on partial restitution processes in kidneys of treated animals three weeks after withdrawal of the toxin. The degree of reparative changes can be characterized as restitution of low intensity. The only difference from the period immediately after withdrawal of the toxin from the feed was that necrotic cells and foci were completely absent from the tubulocytes.

Animals from other experimental groups that were given mixtures containing 2 mg/kg of OTA and various adsorbents followed by additive-free feed for three weeks showed similar changes in the form of regeneration in tubulocytes without changes in the renal tubule lumen. In all three experimental groups, there were no changes observed in the glomeruli, while signs of regeneration were noted in tubulocytes. The difference between the experimental group that received only OTA and the groups that received the toxin with various adsorbents lies in significant restitution in the tubules, without the occurrence of sclerosis and capsule thickening in birds that received OTA and the adsorbents. Furthermore, expansion in glomeruli observed during the consumption of feed contaminated with OTA completely disappeared after three weeks without additives in the feed. There is little information in the available literature on the monitoring of restitution in kidneys after withdrawal of the contaminated feed. Our results are partially consistent with the reports of Santin *et al.* [14] and Garcia *et al.* [12], which refer to the inorganic and mixed adsorbent. Furthermore, there are no data on the comparative testing of three different adsorbents that were used in our experiment with respect to OTA. Therefore, further comparative investigations on the efficacy of different adsorbents and testing different concentrations of OTA, as well as the adsorbents, through various time periods, should be carried out.

## 4. Experimental Section

### 4.1. Animals

During the trial, broiler chickens were kept in metal cages of standard sizes with wire floors. Before allocation of broilers, the experiment room was prepared, *i.e.*, the floor was sanitized and disinfected using biodegradable disinfectant with broad spectrum antimicrobial activity. For the entire trial period, zoohygienic and microclimatic conditions were in full concordance with the technological standards for the given broiler provenience.

The trial was conducted on a total of 160 broilers (1 day old, Ross 308 strain), both sexes, weighing 48.24–48.31 g. Birds were randomly divided into eight groups, resulting in 20 animals per group (control and experimental). The investigation period was 42 days, divided into two phases lasting 21 and 42 days. The groups were kept under standard hygienic and environmental conditions.

### 4.2. Feed Preparation

Broilers were fed a complete diet for chicken fattening, the composition of which was age-dependent during the experiment. Three mixtures of standard composition were used, completely satisfying the nutritional needs of broilers of different ages: Starter (Days 1–21), grower (Days 22–35) and finisher (Days 36–42) [29,30]. Water was provided using manual waterers changed daily. The feeding regime was *ad libitum*. The usual procedures were applied for the sampling and preparation of feeding diet mixtures.

The preparation of contaminated food was performed as previously described [31]. The contaminated diet was prepared using OTA obtained by contamination of corn with *Aspergillus* spp. The toxin producer was the *Aspergillus ochraceus* Wilhelm NRRL 263.67 culture, obtained from the Dutch collection. Conidiospores of *A. ochraceus* were cultivated on potato dextrose-agar substrates, for five days at 27 °C, followed by corn contamination with obtained cultures. Corn contamination lasted 10 days, during which time, full growth of the mold was achieved, resulting in OTA production. The corn with cultures of *A. ochraceus* was kept at a temperature of 15 °C–25 °C during contamination. After 10 days, the corn was dried at 105 °C in a laboratory drying cabinet in order to destroy the mold, ground and used for the contamination of the experimental premixes. HPLC analysis of contaminated corn revealed an OTA concentration of 270 mg/kg. This was used for the inoculation of the broiler diet to the final OTA concentration of 2 mg/kg. The diet was previously tested for the presence of other mycotoxins, and all tests were negative. All procedures and handling of OTA and the contaminated feed were performed with due precautions and the use of protective equipment.

Before artificial contamination with OTA, the feed was tested for the presence of other mycotoxins in order to avoid synergistic toxic effects on broilers. Analyses were carried out using ELISA kits for aflatoxin B1 (Celer^®^ Afla B1, Tecna, Trieste, Italy, limit of detection LoD = 0.5 μg/kg; limit of quantification LoQ = 1 μg/kg), deoxynivalenol (Celer^®^ DON v3, Tecna, Trieste, Italy, LoD = 40 μg/kg; LoQ = 125 μg/kg), trichothecenes (Celer^®^ T2, Tecna, Trieste, Italy, LoD = 15 μg/kg; LoQ = 25 μg/kg), fumonisins (Celer^®^ Fumo, Tecna, Trieste, Italy, LoD = 750 μg/kg; LoQ = 1,000 μg/kg) and zearalenone (MaxSignal^®^ zearalenone ELISA test kit, Bioo Scientific Corp, Austin, TX, USA, LoD = 0.7 μg/kg; LoQ = 1 μg/kg). Samples (*n* = 6), randomly took from the feed sacks, were prepared according to the instructions provided by the manufacturers of the ELISA kits. Optical density was measured at 450 nm using the ELISA reader Thermo Scientific, Thermo (Waltham, MA, USA), model 364. Ascent v1.0 software, Thermo Scientific, Thermo (Waltham, MA, USA) was used for data acquisition and processing. Results for all mycotoxins were below the quantification limits of the methods.

Feed contamination with OTA was accomplished using the standard feed mixer, by adding artificially-contaminated maize (the initial OTA concentration was 270 mg/kg). Mixing was carried out in two batches, the targeted concentration of OTA being 2 mg/kg. Homogeneity of OTA was tested after each mixing using the HPLC-Fl method. Six samples of contaminated feed were analyzed per batch in order to establish mixing efficiency.

Quantitative determination of OTA in the feed was conducted using HPLC with fluorescence detection. The Association of Official Agricultural Chemists method (AOAC, Natural Toxins, 2000) was used for the extraction of the toxin, in the following chromatographic conditions: Mobile phase, 4% CH_3_COOH: acetonitrile = 40:60; flow, 1 mL/min; HPLC column, Waters Symmetry shield 150 × 3.9 mm, 5 μm particle size. The fluorescence detector was set to λex = 334 nm and λem = 460 nm. The obtained retention time of OTA was 3.4 min. The detection limit was 0.5 μg/kg. Table 5 shows the results of each sample analysis and the relative standard deviation of the results.

**Table 5:** Results of the homogeneity testing of feed contaminated with OTA. RSD (%): relative standard deviations.

Batch	Sample 1 (mg/kg OTA)	Sample 2 (mg/kg OTA)	Sample 3 (mg/kg OTA)	Sample 4 (mg/kg OTA)	Sample 5 (mg/kg OTA)	Sample 6 (mg/kg OTA)	RSD (%)
1	1.87	1.90	2.21	1.69	2.31	2.05	11.49
2	2.06	1.88	1.87	2.03	2.22	1.90	6.88

The adsorbents used in the experiments were obtained from their manufacturers. The following adsorbents were used: The inorganic adsorbent was “Minazel-plus” (Patent komerc, Mišićevo, Serbia), zeolite (clinoptilolite) obtained from zeolitic tuff, modified by the addition of organic cations; the organic adsorbent was “Mycosorb” (Alltech, Geneva, IL, USA), esterified glucomannans obtained from yeast cell wall derived from *Saccharomyces cerevisiae* 1026; the mixed adsorbent was “Mycofix-plus” (Biomin, Herzogenburg, Austria), a multicomponent adsorbent consisting of HSCAS, cultures of BBSH 797 microorganisms (DSM 11798, Genus novus of family *Coriobacteriaceae*), enzymes and plant-originating fycofite components.

### 4.3. Experimental Design

The control group of broilers was given feed containing neither the toxin nor adsorbents (C). The first experimental group (E-I) was given feed contaminated with 2 mg/kg of OTA only, while the other three experimental groups received feed with 2 mg/kg of OTA with the addition of the tested adsorbents, inorganic (E-II), organic (E-III) and mixed (E-IV). The other three experimental groups were given feed supplemented with the tested adsorbents, inorganic (Mz), organic (Ms) and mixed (Mf), without the addition of OTA. All adsorbents were mixed in a complete broiler ration at the recommended concentration of 0.2%.

All experimental groups were fed the contaminated diet up to Day 21, after which they were fed the ration without added OTA or adsorbents until the end of the investigation (42nd day). At the end of each week (from Week 1 to Week 6), broilers were weighed individually in order to determine the average weekly body weight. Feed consumption was recorded daily, and the mean was calculated weekly up to the end of the experiment. Feed conversion ratios (FCRs) were calculated for each group at the end of the experiment as kg of feed/kg of gain.

### 4.4. Sample Collection

After the 21st day, 10 animals from each experimental and control group were sacrificed. Samples for pathohistological examination were then taken from the liver and kidneys. Upon completion of the experiment on the 42nd day, the remaining animals were humanely sacrificed, and liver and kidney samples were taken for pathohistological examination.

An autopsy and detailed macroscopic examination were performed immediately after the sacrifice of the animals. Samples of kidney and liver were taken at the autopsy for pathohistological examination. The samples were fixed in a solution of 10% neutral formaldehyde and embedded in paraffin. Tissue samples (5–8 μm) were stained by the hematoxylin eosin technique (HE), as described by Scheuer and Chalk [32]. Liquid chromatography (HPLC) with fluorescence detection was used for the determination of OTA content in feed mixtures. The extraction of toxins from the mixtures was conducted according to the AOAC method.

### 4.5. Statistical Analysis

All values are expressed as means and the standard error of the mean (mean ± SE). Unless stated otherwise, all results were tested using ANOVA and Student’s *t*-test. Differences were considered significant at *p <* 0.05. Statistical analyses were performed using commercial statistical software, GraphPad Prism, Version 4.0 (GraphPad Software, Inc., San Diego, CA, USA). All procedures in the study conformed to Directive 2010/63/EU [33] and were approved by the Ethics Committee, Department of Nutrition and Botany, Faculty of Veterinary Medicine, University of Belgrade.

## 5. Conclusions

In our research, we used higher concentrations of OTA that those that are commonly found in the feed of broilers. By using feed contaminated with OTA at a concentration of 2 mg/kg, we ensured the formation of toxic effects in broilers, and therefore, we were able to recognize the protective effects of the adsorbents.

By observing the overall production results for the entire experiment, it can be concluded that OTA exhibits negative effects, as early as in the first week of consuming the contaminated feed. The intensity of the obtained results is proportional to the length of exposure and the amount of the toxin, as well as the presence or absence of adsorbents in feed. OTA exhibits a prolonged effect, which indicates that it probably possesses a cumulative effect. Significantly lower production results, even after withdrawal of the contaminated feed, strongly supports this claim.

Observed pathoanatomical and pathohistological alterations were less severe in broilers that were fed diet containing both OTA and various adsorbents. However, it was difficult to grade the intensity of differences between those groups. Based on our results, it is evident that all three of the used adsorbents are able to provide significant protection against the toxic effects of OTA in broilers, but this is not sufficient when the concentration of the OTA in feed is 2 mg/kg. It can also be concluded that the adsorbents in the applied concentrations could not completely alleviate the adverse effects of OTA. Furthermore, it is necessary to carry out further research, which would include the use of higher concentrations of adsorbents in the presence of different doses of OTA for a certain period of time.
